# Kidney disease and all-cause mortality in patients with COVID-19 hospitalized in Genoa, Northern Italy

**DOI:** 10.1007/s40620-020-00875-1

**Published:** 2020-10-06

**Authors:** Elisa Russo, Pasquale Esposito, Lucia Taramasso, Laura Magnasco, Michela Saio, Federica Briano, Chiara Russo, Silvia Dettori, Antonio Vena, Antonio Di Biagio, Giacomo Garibotto, Matteo Bassetti, Francesca Viazzi, GECOVID working group, Anna Alessandrini, Marco Camera, Emanuele Delfino, Andrea De Maria, Chiara Dentone, Antonio Di Biagio, Ferdinando Dodi, Antonio Ferrazin, Giovanni Mazzarello, Malgorzata Mikulska, Laura Ambra Nicolini, Federica Toscanini, Daniele Roberto Giacobbe, Antonio Vena, Lucia Taramasso, Elisa Balletto, Federica Portunato, Eva Schenone, Nirmala Rosseti, Federico Baldi, Marco Berruti, Federica Briano, Silvia Dettori, Laura Labate, Laura Magnasco, Michele Mirabella, Rachele Pincino, Chiara Russo, Giovanni Sarteschi, Chiara Sepulcri, Stefania Tutino, Roberto Pontremoli, Valentina Beccati, Salvatore Casciaro, Massimo Casu, Francesco Gavaudan, Maria Ghinatti, Elisa Gualco, Giovanna Leoncini, Paola Pitto, Kassem Salam, Angelo Gratarola, Mattia Bixio, Annalisa Amelia, Andrea Balestra, Paola Ballarino, Nicholas Bardi, Roberto Boccafogli, Francesca Fezza, Elisa Calzolari, Marta Castelli, Elisabetta Cenni, Paolo Cortese, Giuseppe Cuttone, Sara Feltrin, Stefano Giovinazzo, Patrizia Giuntini, Letizia Natale, Davide Orsi, Matteo Pastorino, Tommaso Perazzo, Fabio Pescetelli, Federico Schenone, Maria Grazia Serra, Marco Sottano, Roberto Tallone, Massimo Amelotti, Marie Jeanne Majabò, Massimo Merlini, Federica Perazzo, Nidal Ahamd, Paolo Barbera, Marta Bovio, Paola Vacca, Andrea Collidà, Ombretta Cutuli, Agnese Lomeo, Francesca Fezza Nicola Gentilucci, Nadia Hussein, Emanuele Malvezzi, Laura Massobrio, Giula Motta, Laura Pastorino, Nicoletta Pollicardo, Stefano Sartini, Paola Vacca Valentina Virga, Italo Porto, Giampaolo Bezante, Roberta Della Bona, Giovanni La Malfa, Alberto Valbusa, Vered Gil Ad, Emanuela Barisione, Michele Bellotti, Aloe’ Teresita, Alessandro Blanco, Marco Grosso, Maria Grazia Piroddi, Paolo Moscatelli, Paola Ballarino, Matteo Caiti, Elisabetta Cenni, Patrizia Giuntini, Ottavia Magnani, Samir Sukkar, Ludovica Cogorno, Raffaella Gradaschi, Erica Guiddo, Eleonora Martino, Livia Pisciotta, Bruno Cavaliere, Rossi Cristina, Farina Francesca, Giacomo Garibotto, Pasquale Esposito, Giovanni Passalacqua, Diego Bagnasco, Fulvio Braido, Annamaria Riccio, Elena Tagliabue, Claudio Gustavino, Antonella Ferraiolo, Salvatore Giuffrida, Nicola Rosso, Alessandra Morando, Riccardo Papalia, Donata Passerini, Gabriella Tiberio, Giovanni Orengo, Alberto Battaglini, Silvano Ruffoni, Sergio Caglieris, Mauro Giacomini, Sara Mora

**Affiliations:** 1 Department of Internal Medicine, Clinica Nefrologica Dialisi e Trapianto, University of Genoa, Hospital Policlinico San Martino, IRCCS, Viale Benedetto XV, 16132 Genoa, Italy; 2 Department of Health Sciences, Infectious Diseases Clinic, University of Genoa, Hospital Policlinico San Martino-IRCCS, Genoa, Italy

**Keywords:** Acute kidney injury, Chronic kidney disease, COVID-19, Mortality, Proteinuria

## Abstract

**Background:**

The prevalence of kidney involvement during SARS-CoV-2 infection has been reported to be high. Nevertheless, data are lacking about the determinants of acute kidney injury (AKI) and the combined effect of chronic kidney disease (CKD) and AKI in COVID-19 patients.

**Methods:**

We collected data on patient demographics, comorbidities, chronic medications, vital signs, baseline laboratory test results and in-hospital treatment in patients with COVID-19 consecutively admitted to our Institution. Chronic kidney disease was defined as eGFR < 60 mL/min per 1.73 m^2^ or proteinuria at urinalysis within 180 days prior to hospital admission. AKI was defined according to KDIGO criteria. The primary and secondary outcomes were the development of AKI and death.

**Results:**

Of 777 patients eligible for the study, acute kidney injury developed in 176 (22.6%). Of these, 79 (45%) showed an acute worsening of a preexisting CKD, and 21 (12%) required kidney replacement therapy. Independent associates of AKI were chronic kidney disease, C-reactive protein (CRP) and ventilation support*.* Among patients with acute kidney injury, 111 died (63%) and its occurrence increased the risk of death by 60% (HR 1.60 [95% IC 1.21–2.49] p = 0.002) independently of potential confounding factors including hypertension, preexisting kidney damage, and comorbidities. Patients with AKI showed a significantly higher rate of deaths attributed to bleeding compared to CKD and the whole population (7.5 vs 1.5 vs 3.5%, respectively).

**Conclusion:**

Awareness of kidney function, both preexisting CKD and development of acute kidney injury, may help to identify those patients at increased risk of death.

**Electronic supplementary material:**

The online version of this article (10.1007/s40620-020-00875-1) contains supplementary material, which is available to authorized users.

## Introduction

A novel coronavirus, severe acute respiratory syndrome coronavirus 2 (SARS-CoV-2), was first identified in December 2019 as the cause of a respiratory illness designated coronavirus disease 2019, or COVID-19 [1]. Pulmonary involvement and respiratory failure have been recognized as the main features of COVID-19, but it is now suggested that involvement of other organs, such as heart, kidney and nervous system may occur and influence the clinical course and patient outcome [2].

Renal involvement, defined both as urinary abnormalities and changes in kidney function, has been described among patients with COVID-19 and it might be present in up to 75% of cases [3]. In particular, the development of acute kidney injury (AKI) has been described as a risk factor associated with high in-hospital mortality [4]. Conversely, data are scanty about the combined effect of chronic kidney disease (CKD) and AKI in the COVID-19 population.

In the setting of COVID-19, abnormal kidney function may be a consequence of hemodynamic alterations, enhanced inflammatory status, coagulation abnormalities, organ cross-talk or of direct renal localization of SARS-CoV-2 [5]. Receptors that mediate virus internalization, such as angiotensin-converting enzyme 2 (ACE2) and transmembrane serine protease 2 (TMPRSS2) [6] are abundant in renal tissue. Therefore, it has been hypothesized that SARS-CoV-2 presents a specific renal tropism, which could constitute the biological basis of the common kidney injury in patients affected by COVID-19 [7].

The aim of our study is to define the prevalence of kidney disease and the incidence of acute kidney failure in a large cohort of adult patients with COVID-19. We focused on the determinants of AKI and the impact of both AKI and CKD on mortality.

## Materials and methods

### Study design and cohort

We performed a retrospective, observational study in all adult patients (aged ≥ 18 years) with at least one respiratory sample positive for SARS-CoV-2 by polymerase chain reaction (PCR) admitted to the Policlinico San Martino Hospital, Genoa, Italy between February 25th, 2020, and April 13th, 2020.

All patients able to provide informed consent agreed to participate in the study. A waiver of informed consent for patients unable to provide it themselves was granted in view of the observational nature of the analyses. The study was approved by the Ethics Committee of Liguria Region (CER Liguria, 114/2020-ID 10420) and was carried out in accordance with the principles of the Declaration of Helsinki.

### Definitions and measurements

AKI was defined according to Kidney Disease: Improving Global Outcomes (KDIGO) criteria [8] as an increase in serum creatinine by > 0.3 mg/dl or an increase in serum creatinine to > 1.5 times baseline. We did not consider the urine output criteria to define AKI because of missing data due to the retrospective nature of the study. AKI was calculated at three different time-points: (a) at hospital admission, comparing creatinine with the median value of serum creatinine calculated from all available values within 180 days before admission, (b) within the first week, and (c) after a week of hospitalization, comparing creatinine with values at admission.

The estimated glomerular filtration rate (eGFR) was calculated using the Chronic Kidney Disease Epidemiology Collaboration creatinine equation [9]. CKD was defined as either eGFR < 60 mL/min per 1.73 m^2^ (median value of available data in the 180 days prior to admission) or presence of proteinuria at urinalysis prior to admission. Progression of proteinuria was defined as new onset or increase from the preexisting condition.

For this study, we did not consider hematuria as a reliable marker of renal disease, since data about indwelling urinary catheter were not always available and this might pose a consistent bias in interpretation of urinalysis results.

### Outcomes

The primary outcome considered was the development of AKI. The secondary outcome was overall mortality, both in-hospital and after discharge. Follow-up period of the included patients ranged up to May 16th, 2020. Information about cause of death was obtained from hospital records or death certificates. The causes of death were classified as respiratory failure due to COVID-19 disease or as cardiovascular diseases, cancer, sepsis, thrombosis or bleeding according to the International Classification of Diseases, Tenth Revision.

### Data collection

Collected data regarded comorbidities, chronic medications, vital signs at admission, in-hospital antibiotics, antiviral and anti-inflammatory treatments, mechanical ventilation and laboratory test results at hospital admission. Demographic, clinical and laboratory data were retrieved from electronic medical records and MedInfo, an online database for anonymous and automatic data collection [10], whose use was approved by the CER Liguria August 28th, 2013. The Charlson Comorbidity Index was used as a measure of total comorbidity burden [11].

### Statistical analysis

Normally distributed variables are presented as mean ± SD and compared using an independent or paired *t*-test, as appropriate. Nonparametric continuous variables are presented as median with interquartile range (IQR). Logarithmically transformed values of skewed variables were used for the statistical analysis. Comparisons between groups were made by analysis of variance. Comparisons of proportions were made using the χ^2^-test or Fisher’s exact test, as appropriate. To identify risk factors associated with the development of AKI, we performed a logistic regression model, with adjustment for risk factors that differed between subjects who developed AKI and those who did not. Kaplan–Meier and log-rank test methods were used to estimate and compare survival curves. Cox proportional regression models were used to estimate the hazard ratio (HR) and 95% confidence intervals (CIs) for the relationship between the occurrence of death and the presence of AKI with or without preexisting CKD and several potential confounding factors with biological plausibility. In model 1, age, sex, and Charlson morbidity index are added to AKI as covariates. In model 2, CKD, history of hypertension, treatment with RAAS-I and C-reactive protein (CRP) are also included. In model 3, AKI and CKD are replaced by the four possible combinations of AKI and CKD and treatment with hydroxychloroquine (HCQ) and corticosteroids are also included. Statistical analyses were performed using Stat-View for Windows (version 5.0.1; SAS Institute, Cary, NC, USA). All statistical tests were 2-sided, and a p value < 0.05 was considered statistically significant.

## Results

### Patient presentation

During the study period, 854 patients received a diagnosis of COVID-19 either upon hospital admission or during hospitalization. The final study population consisted of 777 patients (91.0%), for whom data about previous kidney function within the 180 days before COVID-19 diagnosis were available. The baseline characteristics of patients are outlined in Table 1. The mean age was 70 ± 16 years, 58.6% were males, with a proportion of hypertension and diabetes of 49.5% and 15.8%, respectively. Patients showed an at-admission mean creatinine of 0.9 (0.4) mg/dl and proteinuria of 0.30 (0.85) g/l, and the prevalence of preexisting proteinuria and eGFR below 60 ml/min was 50 and 29%, respectively. Results of urinalysis performed within 48 h from admission were available for 552 patients, and 422 (76%) of them showed proteinuria. Historical data from previous urinalysis were available for 55.2% of patients with proteinuria (233/422), and a progression (worsening or new onset) of the preexisting condition of proteinuria was observed in 38% of the cases (Supplemental Fig. 1). In particular, we found progressing proteinuria in 32% of those who developed AKI and in 17% of those who did not (χ^2^ 8.2, p = 0.0042).

**Table 1:** Baseline characteristics of the study cohort on the basis of kidney status

Variable	ALL (N = 777)	No CKD (N = 555)	CKD (N = 222)	*P *value	No AKI (N = 601)	AKI (N = 176)	*P* value
*Demographic characteristics*							*5
Age, years	70 ± 16	66 ± 16	80 ± 12	< 0.001	68 ± 16	76 ± 13	< 0.001
Male sex, %	59	59	56	0.420	57	65	0.038
*Comorbidity*							
Charlson comorbidity index	1.0 (3.0)	0 (2)	2 (3)	< 0.001	1 (3)	1 (2)	0.103
Hypertension, %	49	43.0	65.9	< 0.001	45.7	62.2	< 0.001
Diabetes mellitus, %	16	12	25	< 0.001	15	20	0.092
eGFR < 60 ml/min/1.73 m^2^, %	28				24	45	< 0.001
Proteinuria (≥ 0.3 g/l), %	50	0	81	< 0.001	42	74	< 0.001
Coronary artery disease, %	12	8	23	< 0.001	10	17	0.033
Congestive heart failure, %	11	5	26	< 0.001	9	17	0.002
Cardiac arrhythmia, %	11	7	22	< 0.001	11	14	0.232
Cerebrovascular disease, %	16	11	30	< 0.001	14	25	< 0.001
Hepatic damage, %	2.8	2	4	0.123	3	3	0.895
COPD, %	9	7	13	0.016	7	14	0.006
Solid tumor, %	9	6	16	< 0.001	9	9	0.978
*Treatment history*							
Calcium channel blockers, %	15	13	20	0.005	13	20	0.018
Angiotensin II receptor blockers, %	14	13	19	0.034	15	14	0.705
ACE-inhibitors, %	15	14	20	0.045	14	20	0.055
Oral anticoagulant, %	10	8	16	< 0.001	9	13	0.224
Subcutaneous anticoagulant, %	3.3	2	5	0.045	2	3	< 0.001
Antiplatelet, %	24	19	36	< 0.001	23	28	0.227
Corticosteroids, %	10	8	15	0.004	8	15	0.004
NSAIDs, %	2.8	3	3	0.812	2	4	0.386
*Clinical presentation*							
Fever, %	82	87	71	< 0.001	83	80	0.269
Cough, %	37	43	20	< 0.001	38	31	0.095
Dyspnea, %	49	49	51	0.515	48	55	0.072
Mental confusion, %	13	10	21	< 0.001	11	20	0.001
Temperature, °C	38.2 ± 6.7	38.1 ± 5.7	38.5 ± 8.9	0.447	38.4 ± 7.4	37.7 ± 3.6	0.251
Glasgow coma scale	15 (0)	15 (0)	15 (1)	0.005	15 (0)	15 (2)	< 0.001
Respiratory rate, breaths per min	21 ± 8	21 ± 8	21 ± 10	0.735	20 ± 7	23 ± 11	0.004
Heart rate, beats per min	88 ± 17	89 ± 16	84 ± 18	0.001	87 ± 16	90 ± 19	0.050
Systolic blood pressure, mmHg	131 ± 21	131.6 ± 20.5	128.1 ± 24.0	0.055	132.1 ± 20.0	126.0 ± 25.3	0.002
Diastolic blood pressure, mmHg	75 ± 13	76.4 ± 12.2	70.0 ± 13.4	< 0.001	75.8 ± 12.2	70.8 ± 14.2	< 0.001
O2 saturation, %	95 (6)	95 (5)	95 (7)	0.108	95 (5)	93 (7)	0.012
PaO_2_/FiO_2_ ratio	267 (168)	271 (160)	238 (183)	0.174	281 (165)	202 (175)	< 0.001
*Radiologic findings in Chest X-ray*							
Normal, %	15	14	17	0.353	16	12	0.213
Bilateral pulmonary infiltration, %	33	35	30	0.236	31	40	0.032
Consolidation, %	65	68	60	0.047	65	68	0.387
Pleural effusion, %	8	5	15	< 0.001	6	13	0.005
*Outcomes*							
Acute kidney injury, %	21.9	17.5	35.6	< 0.001			
CRRT, %	2.8	2.7	2.9	0.928	0	11.9	< 0.001
Non-invasive respiratory support, %	31.7	35	23	0.002	28	43	< 0.001
Invasive mechanical respiratory support, %	12.3	15	5	< 0.001	8	27	< 0.001
Cardiovascular events, %	13.3	11	18	0.010	10	26	< 0.001
All cause death, %	35.4	26	59	< 0.001	27	63	< 0.001
Sars-CoV-2-cause death, %	71.9	77	67	0.061	72	71	0.822
*Treatment*							
COVID-specific treatment, %	68.7	73	57	< 0.001	70	66	0.377
Hydroxychloroquine, %	88.1	91	80	< 0.001	90	82	0.027
Hydroxychloroquine, mg/die	720 ± 170	735 ± 157	660 ± 220	< 0.001	720 ± 170	700 ± 200	0.340
Hydroxychloroquine treatment, days	9 ± 4	9 ± 4	9 ± 5	0.235	9 ± 4	9 ± 4	0.367
Tocilizumab, %	31.5	36.6	14.9	< 0.001	31.4	32.0	0.905
Darunavir/Ritonavir	36.6	39.7	27.4	0.024	34.5	44.0	0.102
Corticosteroid, %	92.1	93.9	86.5	0.019	92.2	91.7	0.877
Anticoagulant, %	71.5	71.9	70.2	0.649	69.8	77.0	0.074

Data presented as mean ± standard deviation (SD) or median (IQR) or percentage

*IQR* interquartile range, *CKD* chronic kidney disease, *AKI* acute kidney injury, *COPD* chronic obstructive pulmonary disease, *NSAIDs* nonsteroidal anti-inflammatory drugs, *PaO*_*2*_ arterial oxygen partial pressure, *FiO*_*2*_ fractional inspired oxygen, *CRRT* continuous renal replacement therapy

Compared with patients discharged alive, non-survivors were older, more frequently male, with a history of hypertension, diabetes, cardiac or cerebrovascular disease, chronic obstructive pulmonary disease (COPD), and solid tumor. They were found to be more likely in treatment with angiotensin converting enzyme inhibitors (ACE-is) and with anticoagulant or antiplatelet drugs. Furthermore, they more likely experienced dyspnea, mental confusion, pathologic chest X-ray with interstitial infiltrates or with mono-bilateral pneumonia (Supplemental Table 1).

### Determinants of kidney disease

**Fig. 1:**
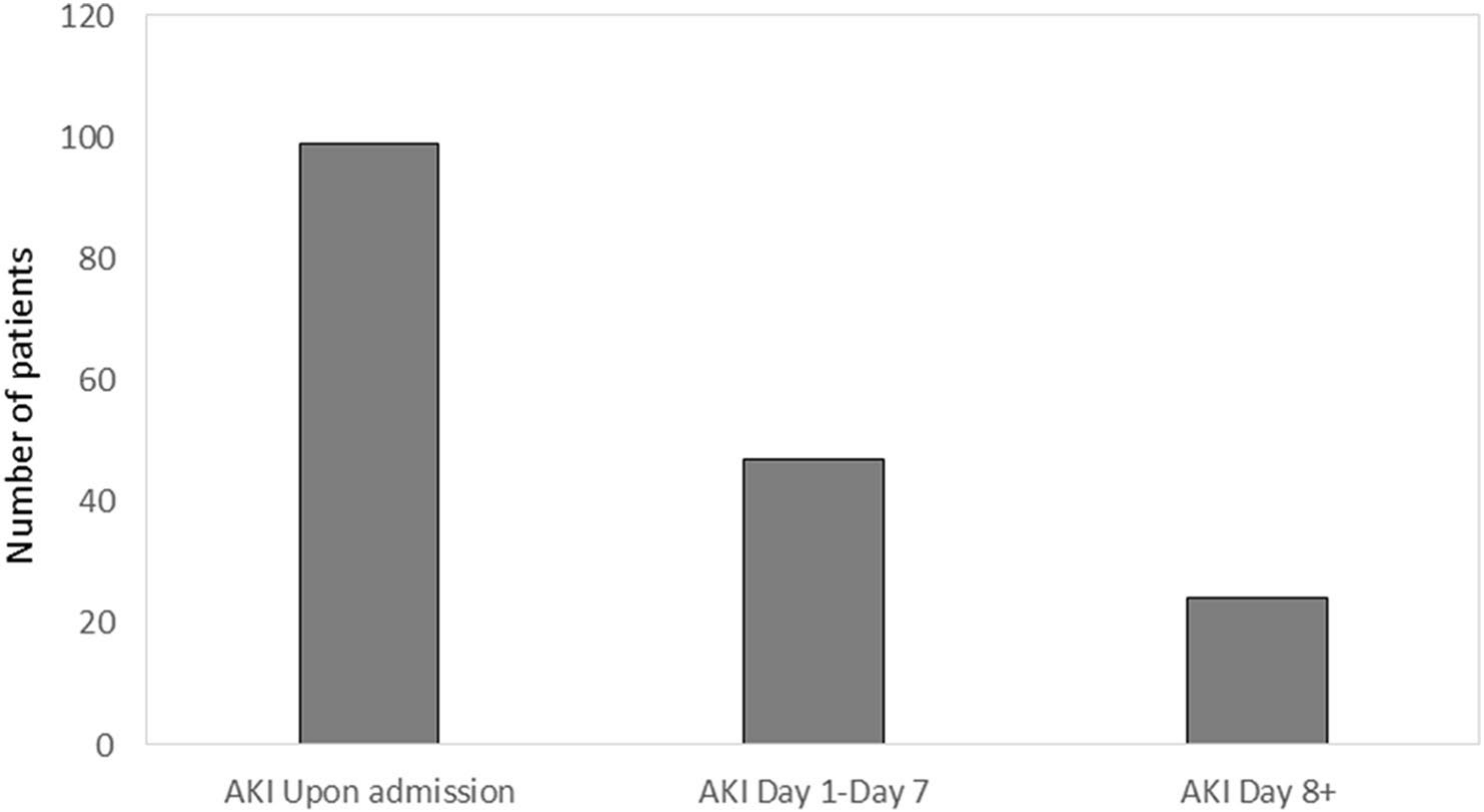
The number of patients with diagnosis of acute kidney injury by length of hospitalization

Among the study population, 176 patients (22.6%) developed AKI at some point during hospitalization. Of these, 79 (45%) showed an acute worsening of a preexisting CKD. The prevalence of CKD in our cohort was 28.6% (n = 222). This proportion was significantly lower when estimated on a historical basis (11.8%) rather than according to previous eGFR and urinalysis data. Different characteristics among included patients with and without pre-existing CKD, as well as with and without AKI are outlined in Table 1. As expected, patients with preexisting CKD were older, more comorbid and were more frequently in treatment for cardiovascular prevention as compared to those without CKD. These subjects showed less frequent respiratory manifestations except for pleural effusion at chest X-ray, and mental confusion. They were less frequently treated with antiviral, anti-inflammatory specific treatment and invasive ventilation and died 2 times more frequently as compared with patients without CKD (Table 1). The timing of the initial development of AKI with respect to hospital admission is displayed in Fig. 1. Most cases developed early in the course, with 58% either arriving with AKI. Twenty-one patients (2.7% out of 777, 12% of the 176 patients with AKI) required continuous renal replacement therapy (CRRT) during the follow-up period. The patients with AKI were older, had more comorbidities and were more frequently in treatment for cardiovascular prevention as compared to those without AKI (Table 1). Mostly, they showed more frequent respiratory manifestations with interstitial infiltrates and pleural effusion at chest X-ray, mental confusion, and a more frequent use of respiratory support and CRRT. There were no differences in the frequency of treatment with anti-inflammatory-specific therapy and anticoagulants as compared to patients without AKI. On the contrary, they less frequently received treatment with HCQ and invasive ventilation and died 2 times more often (Table 1). Most patients received a combination treatment of HCQ with darunavir/ritonavir (284/777; 36.6%). Patients with CKD received antiviral treatment less frequently than those without CKD (27.4% vs 39.7, χ^2^ = 4.956, p = 0.026). On the contrary, darunavir and ritonavir did not show a relationship with AKI (34.5 vs 44.0%, χ^2^ = 2.713, p = 0.102) (Table 1). Using the mortality risk score by Zhao et al. [12] we found that patients with CKD (61.3 vs 38.6, χ^2^ 66.320, p < 0.0001) and patients who developed AKI (70.5 vs 37.6, χ^2^ 70.545, p < 0.0001) showed a significantly higher risk of score mortality ≥ 2 as compared to patients without chronic or acute kidney damage, respectively. Laboratory test results at admission, stratified by kidney status, are outlined in Supplemental Table 2. Both patients with CKD and AKI showed signs of inflammation such as higher leukocytes, procalcitonin and IL6 as compared to those without kidney failure. Only patients with AKI had higher CRP levels and signs of hepatic cytolysis (Supplemental Table 2).

At multivariable analysis, older age and CRP were independently related to the development of AKI. The presence of CKD and non-invasive respiratory support were associated with a two-fold higher risk of AKI, while treatment with HCQ was inversely related with it (Supplemental Fig. 2). On the contrary, treatment with ACE-I or angiotensin receptor blocker (ARB) and IL-6 levels were not significantly related to the development of AKI (HR 1.55 [95% CI 0.99–2.43], p = 0.057; HR 0.91 [95% CI 0.56–1.50], p = 0.705 and HR 1.00 [95% CI 1.00–1.00], p = 0.208, respectively).

### Impact of kidney disease on mortality

During a mean 35 ± 22 days of follow-up, 275 patients died (35%). The occurrence of death was higher among patients who developed AKI compared to those who did not (63 vs 27%, χ^2^ 76.2, p < 0.0001). At Cox univariable analysis AKI was associated with a three-fold higher risk of death (HR 2.9 [95% CI 2.28–3.70], p < 0.0001) and this relationship maintained its statistical significance after adjustment for potential confounding factors in a multivariable model (Table 2).

**Table 2:** Univariate and multivariate cox regression analyses for mortality in hospitalized patients with COVID-19

Risk factors	Univariate Model			Multivariate Model 1			Multivariate Model 2			Multivariate Model 3		
	HR	95% CI	p	HR	95% CI	p	HR	95% CI	p	HR	95% CI	p
Age, years	1.06	1.05–1.07	** < 0.001**	1.06	1.05–1.07	< 0.001	1.07	1.05–1.09	** < 0.001**	1.07	0.05–1.09	** < 0.001**
Male sex	1.40	1.09–1.79	**0.008**	1.47	1.12–1.95	**0.006**	1.27	0.89–1.82	0.182	1.26	0.88–1.81	0.205
Charlson morbidity index	1.18	1.34–1.22	** < 0.001**	1.09	1.05–1.14	** < 0.001**	1.13	1.06–1.21	** < 0.001**	1.13	1.06–1.21	** < 0.001**
AKI	2.90	2.28–3.70	** < 0.001**	2.21	1.69–2.88	** < 0.001**	1.74	1.21–2.50	**0.002**			
CKD	2.83	2.23–3.59	** < 0.001**				1.68	1.17–2.40	**0.005**			
Hypertension, history of	2.30	1.40–2.30	** < 0.001**				0.92	0.62–1.35	0.654	0.91	0.61–1.34	0.621
Home treatment with ACE-inhibitors	1.56	1.15–2.11	**0.004**				1.00	0.65–1.55	0.995	1.00	0.65–1.54	0.988
Home treatment with ARBs	1.11	0.77–1.59	**0.583**				1.31	0.80–2.13	0.281	1.29	0.79–2.11	0.304
C-Reactive protein, each mg/L	1.01	1.00–1.01	** < 0.001**				1.00	1.00–1.01	** < 0.001**	1.00	1.00–1.01	** < 0.001**
Interleukin-6, each pg/mL	1.00	1.00–1.00	**0.052**									
Treatment with hydroxychloroquine	2.75	1.92–3.93	** < 0.001**				0.51	0.33–0.79	**0.002**	0.51	0.33–0.79	**0.002**
Treatment with corticosteroids	1.62	0.91–2.88	0.100									
Invasive mechanical respiratory support	1.19	0.85–1.67	0.304				2.01	1.26–3.40	**0.004**	2.01	1.21–3.35	**0.007**
No kidney disease	ref									ref		
AKI in patients without CKD	5.03	3.55–7.11	** < 0.001**							2.87	1.76–4.67	** < 0.001**
Stable CKD	3.98	2.85–5.55	** < 0.001**							1.89	1.15–3.01	**0.012**
AKI in patients with CKD	3.60	2.65–4.89	** < 0.001**							1.79	1.14–2.81	**0.011**

Bold values are *p * < 0.05

*CI* confidence intervals, *HR* hazard ratio, *AKI* acute kidney injury, *CKD* chronic kidney disease

**Fig. 2:**
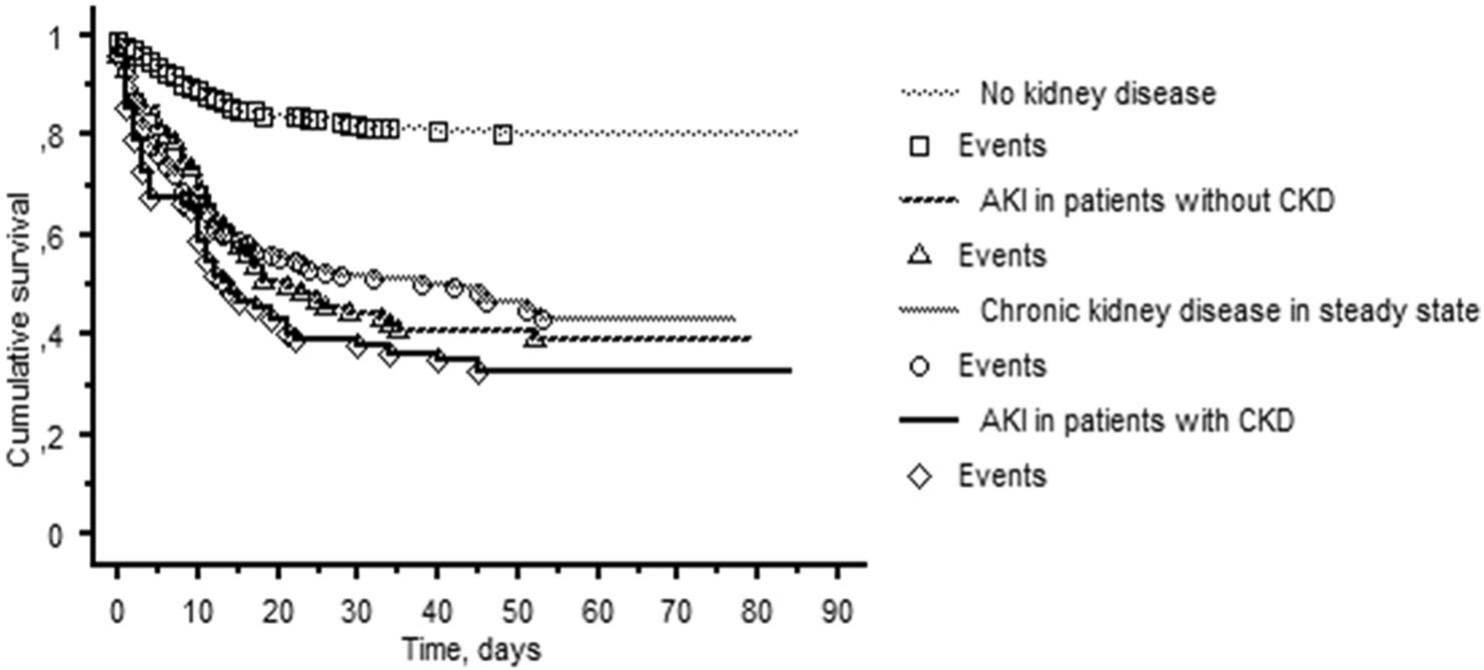
Kaplan Maier curves of survival without death for COVID-19 patients on the basis of chronic kidney disease and/or acute kidney injury. Log Rank test p < 0.0001. *AKI* acute kidney injury, *CKD* chronic kidney disease

When we analyzed the relationship between CKD and/or AKI and death, we found that patients without acute or chronic renal failure showed a significantly lower risk of death as compared to those with CKD or AKI alone or combined (Fig. 2, Log rank p < 0.0001). In particular, patients with CKD complicated by AKI had an almost double risk of death as compared to patients without renal damage (HR 1.79 [95% CI 1.14–2.81], p = 0.011) independently of several confounding factors including age.

Patients with kidney damage showed a similar distribution of the causes of death as compared to the whole population except for bleeding, which was significantly more frequent in AKI as compared to non-AKI patients (7.5% vs 1.3%, χ^2^ 6.7, p = 0.0099). Bleeding represented the cause of death in 1.5% of CKD patients and in 3.8% of the whole population (Supplemental Fig. 3).

## Discussion

Among a large cohort of COVID-19 patients admitted to a Northern-Italy teaching hospital, we observed that about 30% had preexisting CKD and 22% developed AKI. The overall mortality was 35%, but it reached a peak of 63% among patients who developed AKI.

In line with previous reports, mild proteinuria was the most frequent sign of kidney disease [3]. It was observed in 79% of patients and was a worsening of preexisting proteinuria in about 40% of them.

While the study by Williamson et al. [13] provides important information on the epidemiology of COVID-19 and is the first to convincingly demonstrate the importance of CKD as a risk factor for COVID-19 mortality, to our knowledge, this is the first study analyzing the combined effect of CKD and AKI in COVID-19 hospitalized patients in whom preexisting CKD has been specifically investigated on the basis of kidney function (eGFR and urinalysis) within the 180 days before COVID-19 diagnosis. Even when we estimated the prevalence of CKD based on the reported medical history, we observed a significantly higher prevalence of CKD (11.8%) as compared to that described in early reports in China (0.7%) [14] and in Europe (8.5%) [15] on a historical basis. This difference might be explained by the older age of our population and the high prevalence of multiple comorbidities, both well-established risk factors for chronic renal disease [16]. Interestingly, we found that COVID-19 patients with CKD, beyond the classical features of kidney failure, such as lower hemoglobin levels and increased signs of inflammation, showed milder respiratory involvement as compared to those without CKD. Moreover, they less frequently received anti-COVID medications and invasive ventilation, while presenting a doubled mortality rate, when compared with patients without CKD.

With regard to the incidence of AKI among COVID-19 patients, it has been reported with great heterogeneity, mainly because of variable definition criteria and different baseline populations considered [15, 17]. In early reports, AKI was described as a rare event [14], while in larger cohorts of patients from China and the U.S., its incidence varied from 5 to 36% [3, 4, 18]. In a recent meta-analysis including a total of 44 peer reviewed studies (one RCT and 43 observational studies) on 14,866 patients with laboratory-confirmed COVID-19, AKI resulted to be investigated in 15 studies and occurred in 6% of patients (318/4682) [19]. Nevertheless, the Authors raised some doubts about the reliability of data collection and speculated that their summary estimates may represent an underestimation of actual incidence. Interestingly, in a highly selected cohort, excluding the 20 patients on dialysis, 25 (40.3%) out of 62 patients hospitalized in a Nephrology ward in Cremona (Lombardy) developed AKI [20]. Among our study population, we observed an incidence of AKI of 22%. Patients who developed AKI showed a worse clinical and laboratory presentation, characterized by more compromised general conditions with severe respiratory involvement, coagulation abnormalities, and elevation of inflammatory markers, such as CRP, fibrinogen, and IL-6. Nevertheless, when we looked at the determinants of AKI, the strongest parameter maintaining an independent association was CKD. As a matter of fact, one-third of CKD patients developed AKI during hospitalization and about half of AKI patients had a history of CKD. These data are coherent with the evidence, derived from both experimental and clinical studies, supporting the link between AKI and CKD, representing reciprocal risk factors [21, 22].

In different clinical scenarios, it is well-known that local and systemic inflammation may favor AKI [23], but its role in COVID-19 has still to be fully elucidated. The so-called “cytokine storm”, an acute severe systemic inflammatory response, could be one of the most relevant determinants of organ damage during SARS-CoV-2 infection [24]. Data from post-mortem histological findings support the hypothesis that potent inflammation may increase vascular permeability and lead to leukocyte infiltration and erythrocyte aggregation in renal tissue [25]. In our cohort, the only marker of inflammation among the ones studied that retained an association with the development of AKI was CRP. However, inflammation is only one of the determinants of AKI in COVID-19, and growing evidence suggests that multiple mechanisms are involved, including hemodynamic alterations, angiotensin pathway activation, dysregulation of complement, hypercoagulation and also direct virus-mediated injury [26, 27], all factors that were not evaluated in our study due to its retrospective nature.

At variance with previous findings where AKI was more represented among males [28], we did not find a significant difference in sex distribution among our AKI patients. Similarly, the use of ACE-Is or ARBs was not associated with an increased risk of AKI at multivariate analysis.

While acknowledging the limitations of a retrospective study, we found that HCQ, widely used in the present cohort, was independently associated with a reduction in risk of AKI. Interestingly, this finding might have a biological basis, since the anti-inflammatory action of HCQ was postulated to reduce renal damage in some experimental models of acute renal injury [29]. However, opposite findings have been reported [30] and the role of HCQ in the development of AKI has still to be fully elucidated, and larger randomized clinical trials are warranted to address this issue. Use of HCQ in COVID-19 patients is largely debated [31].

Our study offers a longer follow-up to evaluate crude mortality as compared to previous studies that only considered in-hospital mortality [4, 19]. The high mortality rate we observed could be explained, at least in part, by the fact that the participating patients were frail, elderly people with multiple comorbidities [32]. Moreover, the occurrence of AKI at admission or during hospitalization was independently associated with a 74% increase in the risk of death. These findings confirm and extend previous reports [3, 4, 18, 19] and call attention to the need to promptly diagnose and treat and, wherever possible, prevent kidney damage.

To our knowledge, the combined effect of CKD and AKI as risk factors for mortality in COVID-19 has been poorly explored so far and our findings might provide interesting prognostic predictors to be further evaluated in this setting. Several mortality risk scores have been proposed to predict mortality in COVID-19 patients [12, 33, 34], most of which did not include an evaluation of kidney status. In our study cohort, patients with CKD complicated by AKI showed a significantly higher risk of score mortality by Zhao et al. ≥ 2 as compared to patients with only acute or chronic kidney involvement or without kidney damage, respectively (74.7 vs 53.8 vs 67.0 vs 32.5, χ^2^ 131.7, p < 0.0001). These findings suggest a large association between kidney damage and death prediction and recommend the use of renal function for a more accurate assessment of the risk of death in this setting.

As expected, other factors associated with increased death risk were age, comorbidities [35] and increased CRP levels. Of note, IL-6 was not correlated either to the risk of AKI or to the risk of death. We think that these results might be biased by the fact that IL-6 levels were measured only at baseline, while the trend of IL-6 over time could have been more informative [36].

While ACE-Is and ARBs have been hypothesized to both predispose or protect against COVID-19 [37], death rate was not independently correlated with the use of ACE-Is or ARBs in our cohort. On the other hand, multivariable analysis seems to suggest a possible protective effect of HCQ on mortality. Nevertheless, given the retrospective nature of the data and the uneven distribution of HCQ administration among the cohort, these data should be interpreted with caution.

Performing a further observation of specific causes of death in the cohort, death related to bleeding was significantly higher among AKI patients compared to other patients. An overdose of anticoagulants not adjusted according to renal function in patients with underlying coagulation abnormalities may have contributed to this finding [5].

Limitations of our study are its retrospective design, the impossibility to generalize the findings from a single center, the lack of information about the causes of CKD and the lack of follow-up data about possible progression to CKD or normalization of renal function in AKI patients. However, as noted above, this is to the best of our knowledge the first study defining the kidney status on the basis of the median of the creatinine in the 6 months before admission on the whole population. Moreover, preexisting information about proteinuria allowed to calculate the proportion of patients with progressing proteinuria.

## Conclusion

Our data contribute to identify the determinants of AKI and the consequence of the interaction between chronic and acute kidney disease in patients with COVID-19. Large, multicenter, prospective studies with a long-term follow-up are needed to better clarify the impact of renal damage not only during, but also after SARS-CoV-2 infection.

## Electronic supplementary material

Below is the link to the electronic supplementary material.

Supplementary file1 (DOCX 770 kb)

## References

[1] Lai CC, Shih TP, Ko WC, Tang HJ, Hsueh PR (2020). Severe acute respiratory syndrome coronavirus 2 (SARS-CoV-2) and coronavirus disease-2019 (COVID-19): the epidemic and the challenges. Int J Antimicrob Agents.

[2] Yang X, Yu Y, Xu J, Shu H, Xia J, Liu H (2020). Clinical course and outcomes of critically ill patients with SARS-CoV-2 pneumonia in Wuhan, China: a single-centered, retrospective, observational study [published correction appears in Lancet Respir Med. 2020 Apr 8(4):e26]. Lancet Respir Med.

[3] Pei G, Zhang Z, Peng J, Liu L, Zhang C, Yu C (2020). Renal involvement and early prognosis in patients with COVID-19 pneumonia. J Am Soc Nephrol.

[4] Cheng Y, Luo R, Wang K, Zhang M, Wang Z, Dong L (2020). Kidney disease is associated with in-hospital death of patients with COVID-19. Kidney Int.

[5] Ronco C, Reis T (2020). Kidney involvement in COVID-19 and rationale for extracorporeal therapies. Nat Rev Nephrol.

[6] Puelles VG, Lütgehetmann M, Lindenmeyer MT, Sperhake JP, Wong MN, Allweiss L (2020). Multiorgan and renal tropism of SARS-CoV-2 [published online ahead of print, 2020 May 13]. N Engl J Med.

[7] Martinez-Rojas MA, Vega-Vega O, Bobadilla NA (2020). Is the kidney a target of SARS-CoV-2?. Am J Physiol Renal Physiol.

[8] KDIGO Clinical practice guideline for acute kidney injury (2012). Kidney Int Suppl.

[9] Levey AS, Stevens LA, Schmid CH, Zhang YL, Castro AF, Feldman HI (2009). A new equation to estimate glomerular filtration rate [published correction appears in Ann Intern Med. 2011; 155:408]. Ann Intern Med..

[10] Giannini B, Riccardi N, Cenderello G, Di Biagio A, Dentone C, Giacomini M (2018). From Liguria HIV Web to Liguria infectious diseases network: how a digital platform improved doctors' work and patients' care. AIDS Res Hum Retroviruses.

[11] Charlson ME, Pompei P, Ales KL, MacKenzie CR (1987). A new method of classifying prognostic comorbidity in longitudinal studies: development and validation. J Chronic Dis.

[12] Zhao Z, Chen A, Hou W, Graham JM, Li H, Richman PS (2020). Prediction model and risk scores of ICU admission and mortality in COVID-19. PLoS ONE.

[13] Williamson EJ, Walker AJ, Bhaskaran K, Bacon S, Bates C, Morton CE (2020). Factors associated with COVID-19-related death using OpenSAFELY. Nature.

[14] Guan W-J, Ni Z-Y, Hu Y, Liang W-H, Ou C-Q, He J-X (2020). Clinical characteristics of coronavirus disease 2019 in China. N Engl J Med.

[15] Uribarri A, Núñez-Gil IJ, Aparisi A, Becerra-Muñoz VM, Feltes G, Trabattoni D (2020). Impact of renal function on admission in COVID-19 patients: an analysis of the international HOPE COVID-19 (Health Outcome Predictive Evaluation for COVID 19) Registry [published online ahead of print, 2020 Jun 29]. J Nephrol.

[16] Stevens LA, Li S, Wang C, Huang C, Becker BN, Bomback AS (2010). Prevalence of CKD and comorbid illness in elderly patients in the United States: results from the Kidney Early Evaluation Program (KEEP). Am J Kidney Dis.

[17] Wang L, Li X, Chen H, Yan S, Li D, Li Y, Gong Z (2020). Coronavirus disease 19 infection does not result in acute kidney injury: an analysis of 116 hospitalized patients from Wuhan, China. Am J Nephrol.

[18] Hirsch JS, Ng JH, Ross DW, Sharma P, Shah HH, Barnett RL (2020). Acute kidney injury in patients hospitalized with COVID-19. Kidney Int.

[19] Potere N, Valeriani E, Candeloro M, Tana M, Porreca E, Abbate A (2020). Acute complications and mortality in hospitalized patients with coronavirus disease 2019: a systematic review and meta-analysis. Crit Care.

[20] Malberti F, Pecchini P, Marchi G, Foramitti M (2020). When a nephrology ward becomes a COVID-19 ward: the Cremona experience. J Nephrol.

[21] Guzzi F, Cirillo L, Roperto RM, Romagnani P, Lazzeri E (2019). Molecular mechanisms of the acute kidney injury to chronic kidney disease transition: an updated view. Int J Mol Sci.

[22] Chawla LS, Eggers PW, Star RA, Kimmel PL (2014). Acute kidney injury and chronic kidney disease as interconnected syndromes. N Engl J Med.

[23] Rabb H, Griffin MD, McKay DB (2016). Inflammation in AKI: current understanding, key questions, and knowledge gaps. J Am Soc Nephrol.

[24] Coperchini F, Chiovato L, Croce L, Magri F, Rotondi M (2020). The cytokine storm in COVID-19: an overview of the involvement of the chemokine/chemokine-receptor system. Cytokine Growth Factor Rev.

[25] Diao B, Wang CH, Wang RS, Feng ZQ, Tan YJ, Wang HM (2020). Human kidney is a target for novel severe acute respiratory syndrome coronavirus 2 [SARS-CoV-2] infection [preprint posted online April 10, 2020]. medRxiv.

[26] Batlle D, Soler MJ, Sparks MA, Hiremath S, South AM, Welling PA (2020). Acute kidney injury in COVID-19: emerging evidence of a distinct pathophysiology. J Am Soc Nephrol.

[27] Joannidis M, Forni LG, Klein SJ, Honore PM, Kashani K, Ostermann M (2020). Lung-kidney interactions in critically ill patients: consensus report of the Acute Disease Quality Initiative (ADQI) 21 Workgroup. Intensive Care Med.

[28] Guanhua X, Hongbin H, Feng W, Sha T, Huang Q, Li H (2020). Acute kidney injury in patients hospitalized with COVID-19 in Wuhan, China: a single-center retrospective observational study. medRxiv.

[29] Tang TT, Lv LL, Pan MM, Wen Y, Wang B, Li ZL (2018). Hydroxychloroquine attenuates renal ischemia/reperfusion injury by inhibiting cathepsin mediated NLRP3 inflammasome activation. Cell Death Dis..

[30] Edelstein CL, Venkatachalam MA, Dong Z (2020). Autophagy inhibition by chloroquine and hydroxychloroquine could adversely affect acute kidney injury and other organ injury in critically ill patients with COVID-19. Kidney Int.

[31] Das S, Bhowmick S, Tiwari S, Sen S (2020). An updated systematic review of the therapeutic role of hydroxychloroquine in coronavirus disease-19 (COVID-19). Clin Drug Investig.

[32] Guan WJ, Liang WH, Zhao Y, Liang HR, Chen ZS, Li YM (2020). Comorbidity and its impact on 1590 patients with COVID-19 in China: a nationwide analysis. Eur Respir J.

[33] Shang Y, Liu T, Wei Y, Li J, Shao L, Liu M (2020). Scoring systems for predicting mortality for severe patients with COVID-19. EClinicalMedicine..

[34] Bartoletti M, Giannella M, Scudeller L, Tedeschi S, Rinaldi M, Bussini L (2020). Development and validation of a prediction model for severe respiratory failure in hospitalized patients with SARS-Cov-2 infection: a multicenter cohort study (PREDI-CO study) [published online ahead of print, 2020 Aug 8]. Clin Microbiol Infect..

[35] Wu C, Chen X, Cai Y, Xia J, Zhou X, Xu S (2020). Risk factors associated with acute respiratory distress syndrome and death in patients with coronavirus disease 2019 pneumonia in Wuhan, China [published online ahead of print, 2020 Mar 13]. JAMA Intern Med..

[36] Han H, Ma Q, Li C, Liu R, Zhao L, Wang W (2020). Profiling serum cytokines in COVID-19 patients reveals IL-6 and IL-10 are disease severity predictors. Emerg Microbes Infect.

[37] Rico-Mesa JS, White A, Anderson AS (2020). Outcomes in patients with COVID-19 infection taking ACEI/ARB. Curr Cardiol Rep..

