# Supplementary material for: Kidney disease and all-cause mortality in patients with COVID-19 hospitalized in Genoa, Northern Italy
Source: J Nephrol. 2020 Oct 6;34(1):173–83. doi: 10.1007/s40620-020-00875-1 (PMC7538179; doi:10.1007/s40620-020-00875-1)
Supplement: Supplementary file 1 — Supplementary file1 (DOCX 770 kb) [file 40620_2020_875_MOESM1_ESM.docx]

**SUPPLEMENTAL MATERIAL**

**Table of contents**

| **Supplemental Table 1. Baseline characteristics of the study cohort on the basis of length of recovery and survival** |
| --- |
| **Supplemental Table 2. Laboratory characteristics of the study cohort at admission on the basis of kidney status.** |
| **Supplemental Figure 1. Prevalence and changes in proteinuria at admission in hospital.** |
| **Supplemental Figure 2. Multivariate logistic regression analyses of risk factors associated with the development of AKI** |
| **Supplemental Figure 3. Cause of mortality in COVID-19 patients stratified for kidney status** |
| **GECOVID working group list** |

**Supplemental Table 1. Baseline characteristics of the study cohort on the basis of length of recovery and survival**

| *VARIABLE* | ALL (N=777) | Discharged in 7 days  (N=132) | In hospital >7days (N=370) | Non-survivors (N=275) | *P value* |
| --- | --- | --- | --- | --- | --- |
| *Demographic characteristics* | | | | | |
| Age, years | 70 ±16 | 56±17 | 68±114 | 80±11 | <0.001 |
| Male Sex, % | 59 | 45 | 59 | 65 | <0.001 |
| *Comorbidity* |  |  |  |  |  |
| Charlson comorbidity index | 1.0 (3.0) | 0.0 (1.0) | 1.0 (2.0) | 2.0 (3.0) | 0.006 |
| Hypertension, % | 49 | 27 | 48 | 61 | <0.001 |
| Diabetes, % | 16 | 2 | 15 | 23 | <0.001 |
| eGFR<60 ml/min/1.73 m^2^, % | 28 | 7 | 22 | 47 | <0.001 |
| Preexisting proteinuria (≥0.3g/l), % | 50 | 22 | 41 | 65 | <0.001 |
| Coronary artery disease, % | 12 | 4 | 11 | 16 | 0.002 |
| Congestive heart failure, % | 11 | 4 | 6 | 20 | <0.001 |
| Cardiac arrhythmia, % | 11 | 6 | 8 | 19 | <0.001 |
| Cerebrovascular disease, % | 16 | 6 | 11 | 28 | <0.001 |
| Hepatic damage, % | 2.8 | 0.8 | 1.7 | 5.3 | 0.008 |
| COPD, % | 9 | 2 | 8 | 13 | 0.001 |
| Solid tumor, % | 9 | 5 | 7 | 13 | 0.006 |
| *Treatment history* |  |  |  |  |  |
| Calcium channel blockers, % | 15 | 8 | 17 | 14 | 0.058 |
| Angiotensin II receptor blockers, % | 14 | 9 | 17 | 13 | 0.075 |
| ACE-inhibitors, % | 15 | 8 | 14 | 21 | 0.005 |
| Oral anticoagulant, % | 10 | 4 | 8 | 17 | <0.001 |
| Subcutaneous anticoagulant, % | 3.3 | 0.8 | 2.3 | 5.9 | 0.012 |
| Antiplatelet, % | 24 | 11 | 24 | 31 | 0.001 |
| Corticosteroids, % | 10 | 8 | 8 | 14 | 0.031 |
| NSAIDs, % | 2.8 | 0.8 | 3.7 | 2.4 | 0.228 |
| *Clinical presentation* |  |  |  |  |  |
| Fever, % | 82 | 83 | 84 | 80 | 0.376 |
| Cough, % | 37 | 45 | 40 | 28 | <0.001 |
| Dyspnoea, % | 49 | 31 | 50 | 58 | <0.001 |
| Mental confusion, % | 13 | 5 | 6 | 27 | <0.001 |
| Temperature, °C | 38.2 ±6.7 | 38.2 ±6.8 | 38.1 ±6.2 | 38.4 ±7.4 | 0.910 |
| Glasgow coma scale | 15 (0) | 15 (0) | 15 (0) | 15 (2) | <0.001 |
| Respiratory rate, breaths per min | 21 ±8 | 19 ±11 | 20 ±6 | 23 ±9 | 0.001 |
| Heart rate, beats per min | 88 ±17 | 90 ±15 | 88 ±16 | 87 ±18 | 0.308 |
| Systolic blood pressure, mmHg | 131 ±21 | 130.2 ±19.9 | 133.4 ±19.4 | 127.2 ±24.1 | 0.002 |
| Diastolic blood pressure, mmHg | 75 ±13 | 78 ±11 | 76 ±12 | 71.6 ±13.7 | <0.001 |
| O_2_ saturation, % | 95 (6) | 97 (3) | 95 (5) | 92 (8) | <0.001 |
| PaO_2_/FiO_2_ ratio | 267 (168) | 371 (95) | 271 (148) | 211 (149) | <0.001 |
| *Radiologic findings in chest radiograph* | | | | | |
| Normal, % | 15 | 36 | 13 | 8 | <0.001 |
| Bilateral pulmonary infiltration, % | 33 | 22 | 34 | 38 | 0.012 |
| Consolidation, % | 65 | 50 | 68 | 69 | 0.001 |
| *Laboratory Characteristics* |  |  |  |  |  |
| Hemoglobin, g/dL | 13.0 ±2.2 | 14.0 ±1.7 | 13.1 ±2.0 | 12.4 ±25.1 | <0.001 |
| White blood cell count, x10^9^/L | 7.653 ±4.512 | 6.950 ±2.593 | 7.242 ±4.385 | 8.831 ±5.110 | <0.001 |
| C-reactive protein, mg/L | 70 (98) | 18 (37) | 65 (93) | 103 (108) | <0.001 |
| Procalcitonin, ng/mL | 0.15 (0.35) | 0.04 (0.08) | 0.12 (0.23) | 0.35 (0.79) | <0.001 |
| Interleukin-6, pg/mL | 39 (62) | 13 (14) | 35 (45) | 75 (98) | 0.220 |
| Creatinin, mg/dL | 0.9 (0.4) | 0.8 (0.3) | 0.9 (0.3) | 1.1 (0.8) | <0.001 |
| eGFR, ml/min/1.73m^2^ | 73 (39) | 88 (33) | 81 (35) | 55 (41) | <0.001 |
| Urea, mg/dL | 54 ±43 | 31 ±15 | 44 ±31 | 78 ±55 | <0.001 |
| Proteinuria*, % | 71 | 64 | 75 | 69 | 0.022 |
| Proteinuria, g/L | 0.30 (0.85) | 0.00 (0.30) | 0.30 (0.85) | 0.30 (0.70) | <0.001 |

* Among the 552 urinalysis available within 48 hours from admission

Data presented as mean ± standard deviation (SD) or median (IQR) or percentage.
Abbreviations: IQR, Interquartile Range; CKD, chronic kidney disease; COPD, Chronic obstructive pulmonary disease; PaO_2,_ arterial oxygen partial pressure; FiO_2_, fractional inspired oxygen; eGFR, estimated glomerular filtration rate.

**Supplemental Table 2. Laboratory characteristics of the study cohort at admission on the basis of kidney status.**

| *LABORATORY VALUES* | no CKD (N=555) | CKD (N=222) | P value |  | no AKI (N=601) | AKI upon admission (N=99) | AKI within  7 days of H (N=47) | AKI over  7 days of H (N=30) | P value for trend | P value* |
| --- | --- | --- | --- | --- | --- | --- | --- | --- | --- | --- |
| Hemoglobin, g/dL | 13.5 ±1.9 | 11.7 ±2.3 | <0.001 |  | 13.1 ±2.1 | 12.6 ±2.7 | 12.3 ±2.5 | 13.1 ±2.2 | 0.013 | 0.023 |
| White blood cell count, x10^9^/L | 7.428 ±4.453 | 8.219 ±4.618 | 0.028 |  | 7.358 ±4.465 | 9.161 ±4.719 | 8.133 ±4.361 | 7.860 ±4.120 | 0.003 | <0.001 |
| Platelets count, x10^9^/L | 215.532 ±92.784 | 203.809 ±107.200 | 0.130 |  | 213.696 ±98.069 | 207.541 ±88.067 | 195.234 ±97.083 | 224.067 ±108.532 | 0.411 | 0.524 |
| Alanine aminotransferase, U/L | 48 ±79 | 30 ±32 | 0.001 |  | 44 ±71 | 42 ±80 | 36 ±33 | 47 ±35 | 0.855 | 0.746 |
| Aspartate aminotransferase, U/L | 49 ±69 | 4 ±42 | 0.293 |  | 46 ±62 | 63 ±85 | 40 ±27 | 56 ±43 | 0.127 | 0.035 |
| International Normalized Ratio | 1.19 (0.18) | 1.19 (0.24) | 0.265 |  | 1.18 (0.18) | 1.24 (0.18) | 1.22 (0.24) | 1.25 (0.24) | 0.062 | 0.098 |
| D-dimer, mg/L | 948 (974) | 1435 (2481) | 0.327 |  | 895 (984) | 1576 (2229) | 1564 (1913) | 1118 (756) | <0.001 | <0.001 |
| Creatine kinase, U/L | 101 (136) | 90 (141) | 0.611 |  | 137 (261) | 115 (112) | 95 (135) | 39 (62) | 0.054 | 0.009 |
| Fibrinogen, g/L | 5.8 ±2.0 | 5.2 ±1.9 | 0.001 |  | 5.6 ±1.9 | 6.0 ±2.0 | 5.6 ±2.4 | 6.8 ±2.2 | 0.006 | 0.059 |
| Lactate dehydrogenase, U/L | 334 ±154 | 324 ±161 | 0.426 |  | 318 ±144 | 379 ±179 | 358 ±163 | 405 ±214 | <0.001 | <0.001 |
| C-reactive protein, mg/L | 70 (98) | 70 (98) | 0.702 |  | 61 (94) | 105 (126) | 78 (119) | 105 (156) | <0.001 | <0.001 |
| Procalcitonin, ng/mL | 0.12 (0.26) | 0.29 (0.76) | 0.003 |  | 0.11 (0.23) | 0.49 (1.90) | 0.21 (0.32) | 0.36 (0.50) | <0.001 | 0.373 |
| Interleukin-6, pg/mL | 36 (56) | 56 (90) | 0.009 |  | 34 (55) | 76 (117) | 56 (60) | 59 (65) | <0.001 | <0.001 |
| Creatinine, mg/dl | 0.9 (0.3) | 1.4 (0.8) | <0.001 |  | 0.9 (0.4) | 1.8 (1.2) | 1.0 (0.4) | 1.0 (0.3) | <0.001 | <0.001 |
| eGFR, ml/min/1.73m^2^ | 82 (28) | 44 (31) | <0.001 |  | 79 (32) | 29 (23) | 58 (39) | 73 (30) | <0.001 | <0.001 |
| Urea, mg/dl | 43 ±32 | 83 ±55 | <0.001 |  | 43 ±27 | 120 ±68 | 63 ±43 | 44 ±16 | <0.001 | <0.001 |
| Proteinuria, g/l | 0.30 (0.85) | 0.30 (0.70) | <0.001 |  | 0.30 (0.85) | 1.00 (0.70) | 0.30 (0.74) | 1.00 (0.70) | 0.110 | 0.074 |
| Albumin, g/L | 2.8 ±0.6 | 3.0 ±0.6 | 0.015 |  | 2.8 ±0.6 | 2.7 ±0.7 | 2.8 ±0.6 | 2.5 ±0.7 | <0.001 | 0.011 |

*** AKI upon admission vs no AKI**Data presented as mean ± standard deviation (SD) or median (IQR) or percentage.
Abbreviations: IQR, Interquartile Range; CKD, chronic kidney disease; AKI, acute kidney injury; H, hospitalization; eGFR, estimated glomerular filtration rate.

**Supplemental Figure 1. Prevalence and changes in proteinuria at admission in hospital.**

a) Prevalence of proteinuria and b) changes in proteinuria during hospitalization.


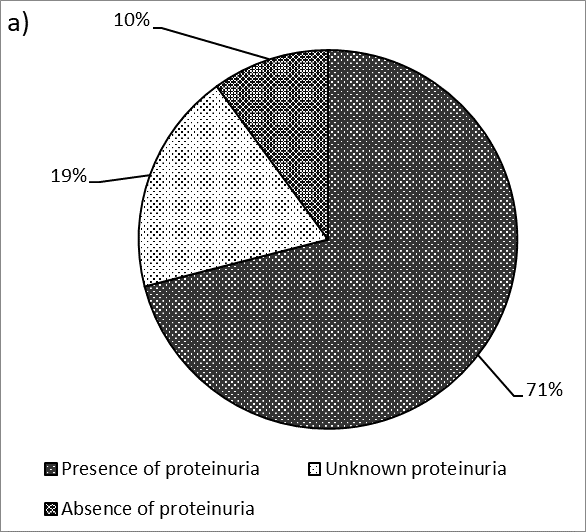

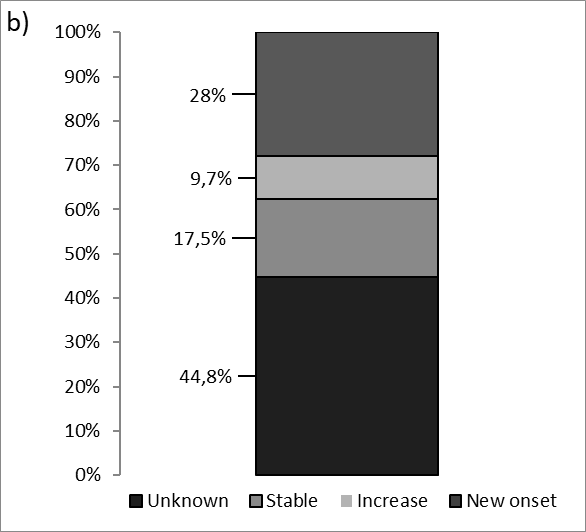


**Supplemental Figure 2. Multivariate logistic regression analyses of risk factors associated with the development of AKI**


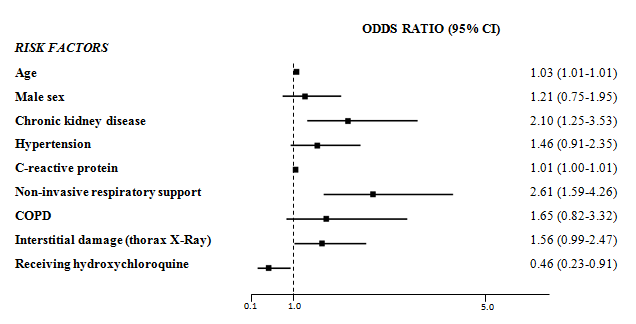


Abbreviations: COPD, Chronic obstructive pulmonary disease; ACE, angiotensin-converting enzyme; ARB, Angiotensin II receptor blockers.

**Supplemental Figure 3. Cause of mortality in COVID-19 patients stratified for kidney status**


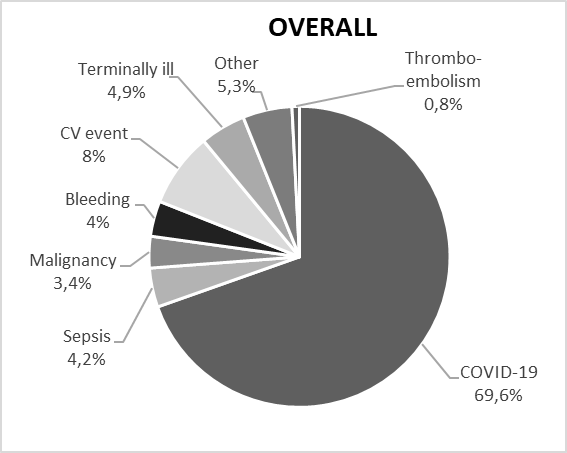

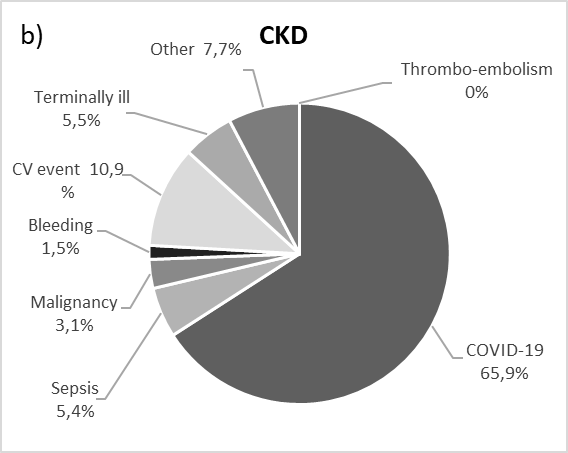

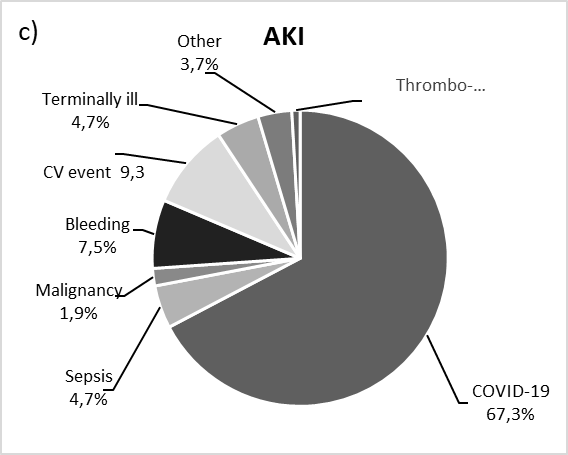


a)

Causes of death in a) overall study population, b) CKD patients and c) patients who developed AKI.

Abbreviations: CKD, chronic kidney disease; AKI, acute kidney injury, CV, cardiovascular.
